# Salivary gland secretory carcinoma presenting as a cervical soft tissue mass: a case report

**DOI:** 10.1186/s13256-024-04364-y

**Published:** 2024-02-05

**Authors:** Parisa Mokhles, Alireza Sadeghipour, Pegah Babaheidarian, Saleh Mohebbi, Zahra Keshtpour Amlashi, Mohammad Hadi Gharib, Mohammad Saeid Ahmadi, Zeinab Khastkhodaei

**Affiliations:** 1 Faculty of Medicine, School of Medicine, Iran University of Medical Sciences, Hemmat Highway, Tehran, Iran; 2 Skull Base Research Center, School of Medicine, The Five Senses Health Institute, Iran University of Medical Sciences, Tehran, Iran; 3 Cancer Research Center, Hamadan University of Medical Sciences, Hamadan, Iran; 4 Golestan University of Medical Sciences, Gorgan, Iran; 5 Hamadan University of Medical Sciences, Hamadan, Iran; 6 Institute of Physiology, University Medicine of the Johannes Gutenberg, Mainz, Germany

**Keywords:** Secretory carcinoma, Salivary glands, Mammary analogue secretory carcinoma

## Abstract

**Background:**

Secretory carcinoma (SC) has been described as a distinct salivary gland tumor in the fourth edition of the World Health Organization (WHO) classification of head and neck tumors. SC is generally considered as a slow-growing low-grade malignant tumor, while several cases have been reported with high-grade features, and even metastases in the literature up until now. In this article, a soft tissue SC case is discussed with high-grade microscopic features and neural invasion. A review of the salivary gland SC cases with aggressive behavior is also debated.

**Case presentation:**

A 65-year-old Caucasian man presented with a left neck mass for the past six months. The imaging studies demonstrated a very large cystic cervical mass (46 × 23 mm) with papillary projections in the anterolateral aspect of the left neck zone Vb. He underwent left radical neck dissection (level I-V) and was followed up for 12 months with the diagnosis of Secretory carcinoma.

**Conclusion:**

Although SC generally has a good outcome, multiple recurrences and unusual metastases may occur, which should be considered by either the pathologists or clinicians.

## Background

SC is generally considered as a low-grade salivary gland tumor, characterized by the morphological resemblance to mammary analogue secretory carcinoma (MASC) [1]. Due to the rarity and novelty of its concept, the incidence is still unknown, but it has been estimated that SC accounts for less than 0.3% of all salivary gland tumors [2].

Before Skalova's description, this entity was confused with various other low-grade salivary gland tumors such as adenocarcinoma (NOS), mucin-producing signet ring adenocarcinoma, mucoepidermoid carcinoma (MEC), and acinic cell carcinoma (ACC) [2–4]. SC has been most falsely reported as ACC in the majority of cases [5, 6]. Although chromosomal translocation causing ETV6 and NTRK3 gene fusion is characteristic of this neoplasm, some authors have supported the use of morphological features and immune histochemistry studies as convenient tools for the diagnosis [3, 5, 7–9].

SC typically presents in patients between the ages of 40–60 with slight male predominance. The most common presentation is a slow-growing, painless nodule [3]. Seventy percent of SC tumors are found in the major salivary glands (predominantly the parotid gland), and others arise from minor salivary glands [10].

Although SC typically presents as a well-demarcated nodule with a low to intermediate-grade histopathologic feature, occasionally high-grade transformation (HGT) has been reported which tends to demonstrate a more aggressive natural history and ultimately a poor prognosis [11]. Histologically, a HGT is characterized by atypical tumor cells with a solid architecture, increased mitotic figures, and foci of comedo-type necrosis [12].

In general, the treatment of choice for SC is similar to the standard of care for other salivary gland carcinomas. Radical surgical resection is the modality of choice for low-grade SC and local radiation therapy can be considered for larger tumors or those with positive margins or perineural invasion. In the case of distant metastasis, systemic chemotherapy may be used [13]. To the best of our knowledge, there are about 39 reported SC cases who undergone recurrence after therapy. At most 18 cases with distant metastasis have been reported up to now (Table 1), 14 to the lung and pleura, 3 to the axial bones [14, 15], and only one with cervical adipomuscular tissue metastasis [3].

**Table 1:** Summery of SC case reports with aggressive behavior

Author’s name/year	Case No	Age (mean)	Male/ Female	Location	Size (cm)	IHC (positive)	Neural invasion/HF *	Metastasis	Recurrence/ Death
Skálová A (2010) [17]	16	46	M/F = 9/7	Parotid (13) Minor glands (3)	2.1	S100 Vimentin STAT5a Muc1&4 Mammaglobin	Not seen	CN*(2) Lung (1)	Present (4) Died (2)
Seethala (2012) [16]	36	45.7	M/F = 1.4/1	Parotid (26) Non parotid (10)	1.9–2.5	HMWK S100 Vimentin	Not seen	CN (4)	Present (3)
Connor (2012) [11]	7	40	M/F = 6/1	Oral cavity (4) Parotid (2) Sub mandibular gland (1)	1.86	HMWK S100 Vimentin Ck19	Present (3)	Not reported	Not reported
Ito (2012) [26]	1	37	F	Parotid	2.0	HMWK S100 Vimentin	Present	Not reported	Not reported
Min jung jung (2013) [4]	13	46.4	M/F = 8/5	Parotid (11) Non-parotid (2)	1.77 (0.7–2.5)	S-100 GCDFP (2/13)	Not seen HF** (1)	Not reported	Present (3)
Bishop (2013) [7]	11	56	M/F = 4/7	Oral cavity (9) Sub mandibular (2)	0.9	Mammaglobin S100	Present (1)	Not reported	Present (1)
Melin-Aldana (2013) [27]	1	14	F	Parotid	3	Not reported	Not seen	PPN***	Not reported
Wenyie Luo (2014) [28]	1	41	F	Hard palate	1.8	Ck7, Ck8/18, S100, BAF47, Vimentin	Present HF	CN	Not reported
Skálová A (2014) [3]	3	63	All M	Parotid (3)	4–8	S100 EGFR and β-catenin (in high grade component) Cyclin-D1	Present (1) HF (3)	Cervical adipose tissue (1)	Present (1) All 3 died
Majeweska (2014) [23]	7	51.4	M/F = 5/2	Parotid:6 Hard palat:1	2.8	S100 Mammaglobin Cytokeratin CK7, CK8 STAT5a Vimentin	Present (2) HF (2)	CN (3) Lung (1) Bone (1)	Present (2) Died (2)
Todd M (2015) [25]	14	54.5	M/F = 6/8	Parotid:9 Sub mandibular:1 Thyroid:1 Lip:2 Hard palate:1	–	Mammaglobin S100	Present (1) HF (1)	CN (2) Lung (1)	Present (1)
Monica L (2015) [29]	4	50.5	M/F = 1/3	Parotid:1 Buccal mucosa:2 Sub mandibular:1	2.6	Mammaglobin S100 STAT5	Not seen HF (1)	CN (1)	Not reported
Nasir Udin (2016) [30]	11	27.5	M/F = 7/5	Parotid:7 Sub mandibular:3 Buccal:1	4.4	S100 EMA CK7/19	Present (1)	CN (2)	Present (3)
Kensuke Suzuki (2017) [31]	1	74	–	Un known origin	-	S100 GATA3	Not seen HF	CN Lung	Not reported
Baghai (2017) [15]	10	46.9	M/F = 6/4	Parotid:9 Minor gland:1	3	Mammaglobin S100	Present (1) HF (2)	CN (3) Bone (1)	Present (3) Died (2)
Nicole A (2017) [13]	1	44	M	Parotid	3.5	S100 Ck7 Mammaglobin	Present HF	Pleura TN****	Died
Huang MD (2017) [20]	1	62	F	Bronchus (minor glands)	8.5	Mammaglobin S100 (focal)	Not seen	CN	Not reported
David Forner (2018) [24]	13	54	M = F	Parotid (9) Minor salivary gland (3) Submandibular (1)	Small in size	S100 Vimentin Mammaglobin Ck7	Present (3) HF (1)	CN (1) Lung (1)	Present (1)
Rooper (2018) [32]	1	59	F	Submandibular	4.7	S100 Mammaglobin	Not seen HF	CN Lung Bone	Present
Numano (2019) [19]	1	65	F	Parotid	6.9	S100 GATA3 AE1/AE3 EMA GCDFP(very focal) Muc 1 (focal)	Present HF	CN Lung	Present Died
Jingjing Sun (2019) [21]	59	63.7 in 3HF 43.2 in conventional	M/F (In conventional) = 4.9/1 All 3 HF were male	Parotid (49) Submandibular (2) Minor glands (8)	3.7 HF 2.2 conventional	Ki 67	Present (5) HF (3)	CN (1) Lung (4)	Present (7) Died (1)
Takabatake (2020) [12]	1	54	M	Hard palate	2.5	S100 Vimentin AE1/3 EMA Mammaglobin	Not seen HF	CN	Not reported
Delima (2021) [33]	1	60	M	Secretorty carcinoma ex pleomorphic adenoma Submandibular gland	–	Mammaglobin S100 (focal) Ck7 Adipophilin	Not seen	CN	Not reported
Jingjing Sun (2021) [1]	23	45	M/F = 13/10	Parotid (21) Submandibular (22)	2.6 (0.8–4.8)	S-100 Mammaglobin CK7 GATA3	Not seen	CN (5) Lung (2)	Present (6) Died (1)
Kensuke Suzuki (2022) [22]	2	37&61	Both M	Parotid (2)	1	S100 STAT5 Mammaglobin GATA3 EGFR B-Catenin	Not seen HF (1)	CN (1)	Present (1)

*CN* Cervical lymph nodes

*HF* High grade features

*PPN* Para parotid nodes

*TN* Thoracic nodes

Herein a case of SC with HGT is discussed, presenting as a soft tissue neck mass in a patient with a past medical history of ipsilateral parotidectomy many years ago without available documentation. In addition, we review the literature of SC cases with aggressive behavior in order to clarify its clinical and pathological characteristics.

## Case presentation

A 65-year-old Caucasian man was referred to the Department of Otolaryngology because of a neck mass for the past six months. He reclaimed a left-side total parotidectomy surgery, without available documentation, more than ten years ago. He didn’t confess to any adjuvant therapy and was not under any follow-up. The initial physical exam revealed a bulging mass on the left posterior triangle of the neck which was non-tender, soft, and irregular on palpation. There was also another small subcutaneous lesion in the parotid masseteric region on the same side, and facial neurological examinations were normal.

**Fig. 1:**
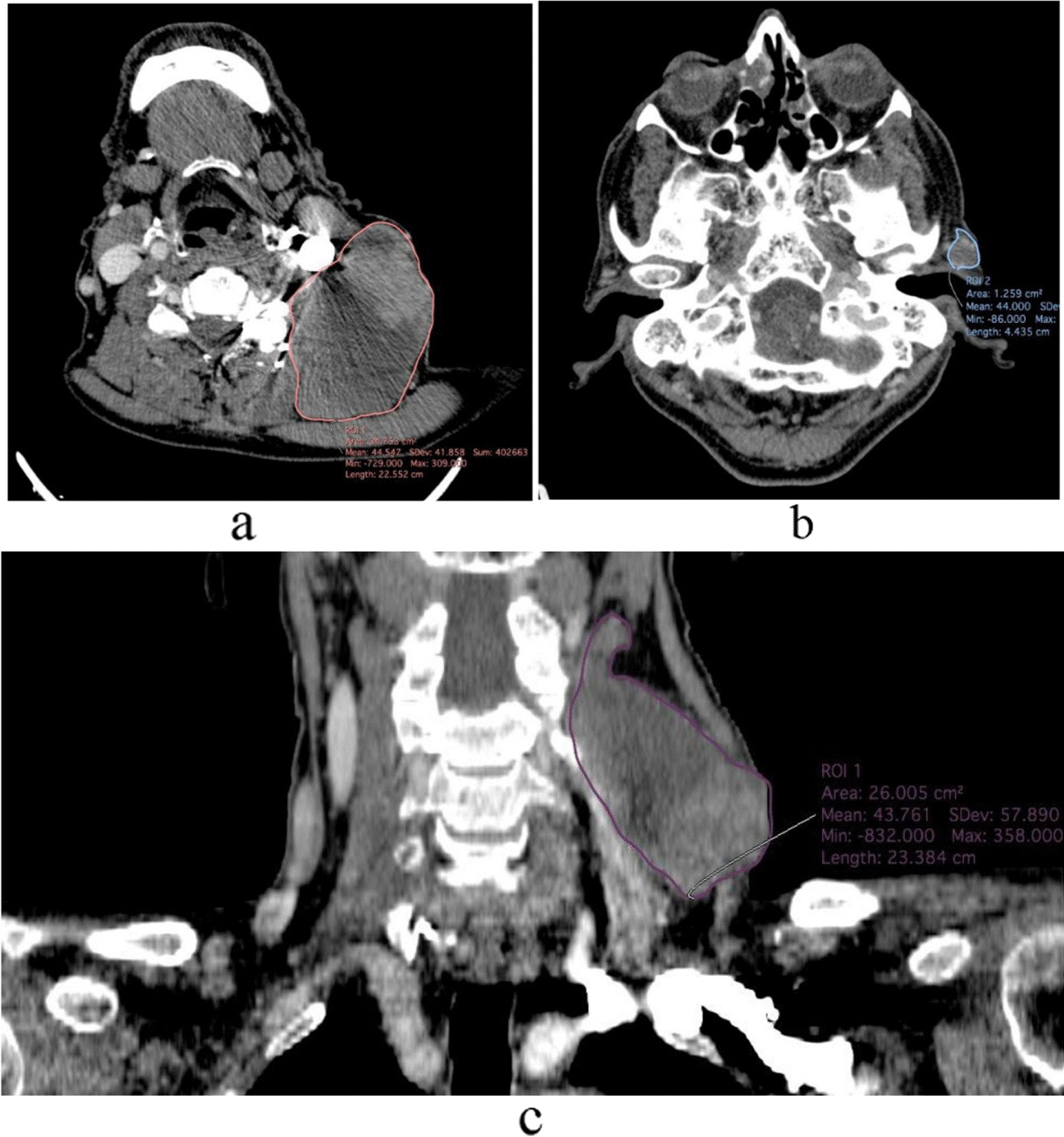
Post contrast neck computed tomography scan; axial and coronal reformat images, demonstrates a very large cystic cervical mass (46 × 23 mm) with papillary projections in its antero-lateral aspect, in left neck zone Vb (**a**, **c**). Moreover, there is a small heterogeneously enhancing nodule with small central cystic component in left pre-auricular region, in the site of prior parotidectomy (**b**)

Post-contrast neck computed tomography (CT) scan; demonstrated a very large cystic cervical mass (46 × 23 mm) with papillary projections in the antero-lateral aspect of the left neck zone Vb (Fig. 1a, c). Moreover, there was a small heterogeneously enhancing nodule with small central cystic components in the left pre-auricular region (at the site of the prior surgical bed) (Fig. 1b). In order to complete the restaging workup, a spiral lung CT was taken, which showed no distant metastases.

Ultrasound-guided fine needle aspiration of the neck mass was done, and microscopic examinations revealed a hyper-cellular smear with a bloody background, composed of large polygonal oncocytic cells with abundant cytoplasm, round to oval large nuclei and distinct nucleoli, without mitotic activity, arranged in sheets, cohesive clusters, and isolated cells. These findings were more in favor of an oncocytic carcinoma.

After general anesthesia, the patient underwent left radical neck dissection (level I-V) and revision parotidectomy procedure, because of the probability of metastasis and recurrence of the previous surgery site, as well as, the adhesion of the remnant parotid tail to the submandibular gland.

**Fig. 2:**
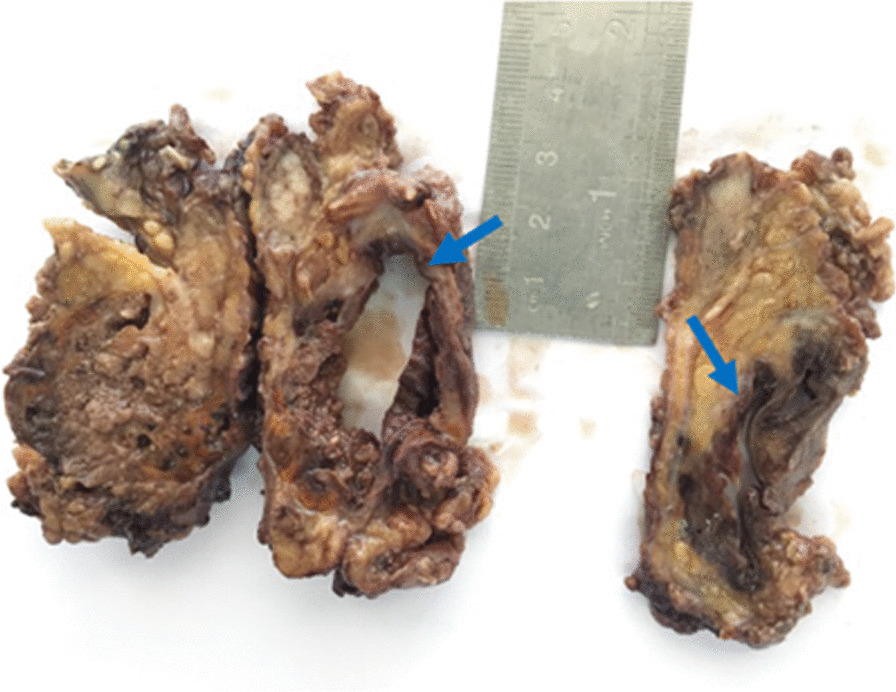
Grossly cut sections of the main lesion were tan-brown and heterogeneous, with hemorrhagic foci, containing a centrally-located hemorrhagic cystic space (showed by blue arrows)

Grossly the main specimen consisted of multiple fragments of tan-brown rubbery tissues, aggregating to about (13.5 × 6.5×3cm). On cut sections, the mass was friable and heterogeneous, with hemorrhagic foci, containing a centrally-located (3.7 × 1.5×1cm) hemorrhagic cystic space (Fig. 2).

**Fig. 3:**
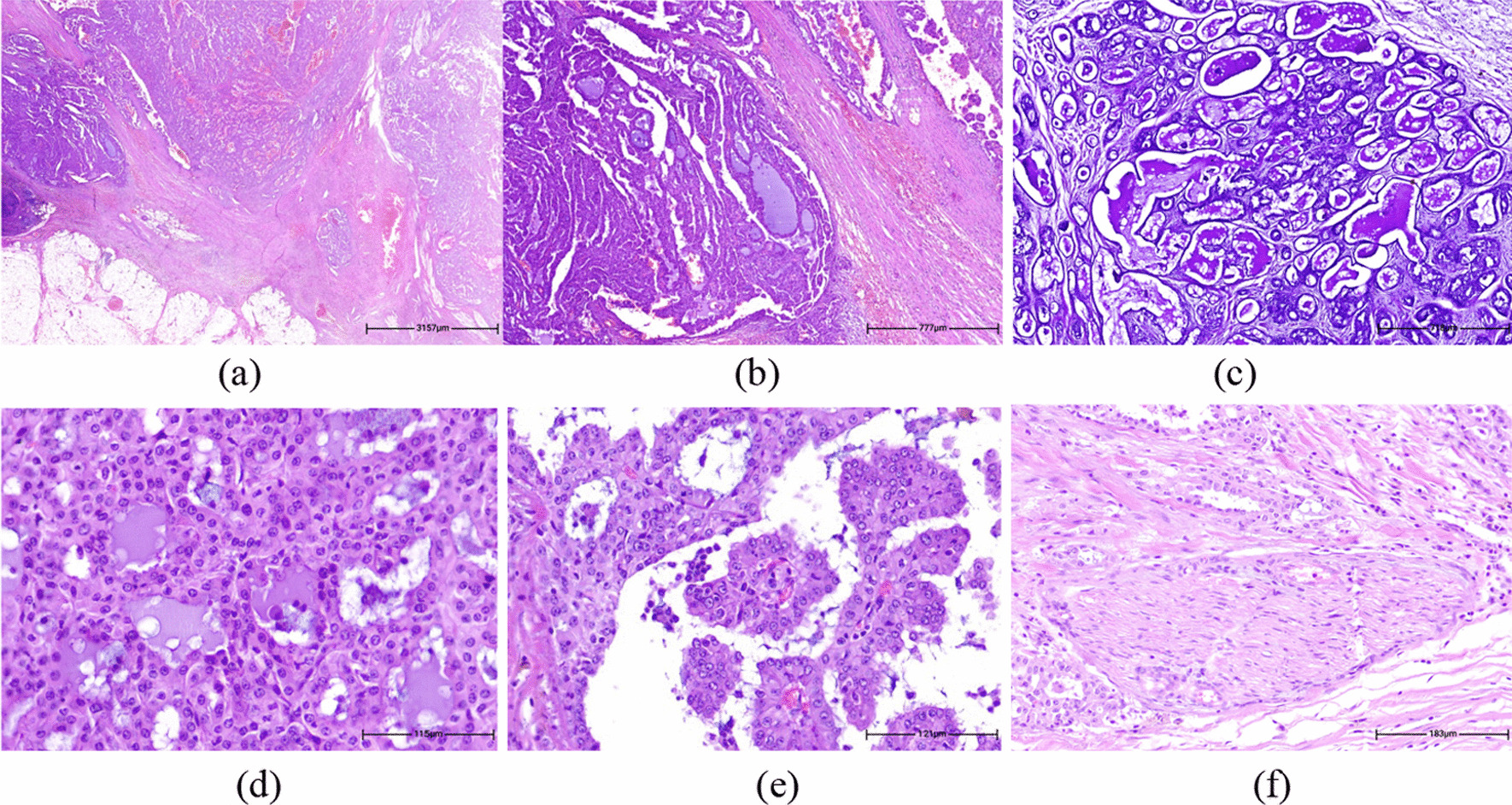
Histopathology sections reveals a multi-nodular proliferation of neoplatic cells, infiltrating adipo-muscular tissue (**a** H&E. ×40). Variable architectural patterns are noted, including of papillary, cribriform, closely packed micro-cysts and tubules (**b** H&E. ×100). Micro-cysts and tubules contain abundant Periodic acid Schiff positive and diastase resistant bubbly secretions (**c** H&E. ×200). Tumor cells have eosinophilic and vacuolated cytoplasm with high N/C ratio, relatively monomorphic round vesicular nuclei and small distinctive nucleoli (**d**, **e** H&E. ×400). Foci of high-grade features, including desmoplastic stromal reaction, and perineural invasion are noted (**f** H&E. ×200)

**Fig. 4:**
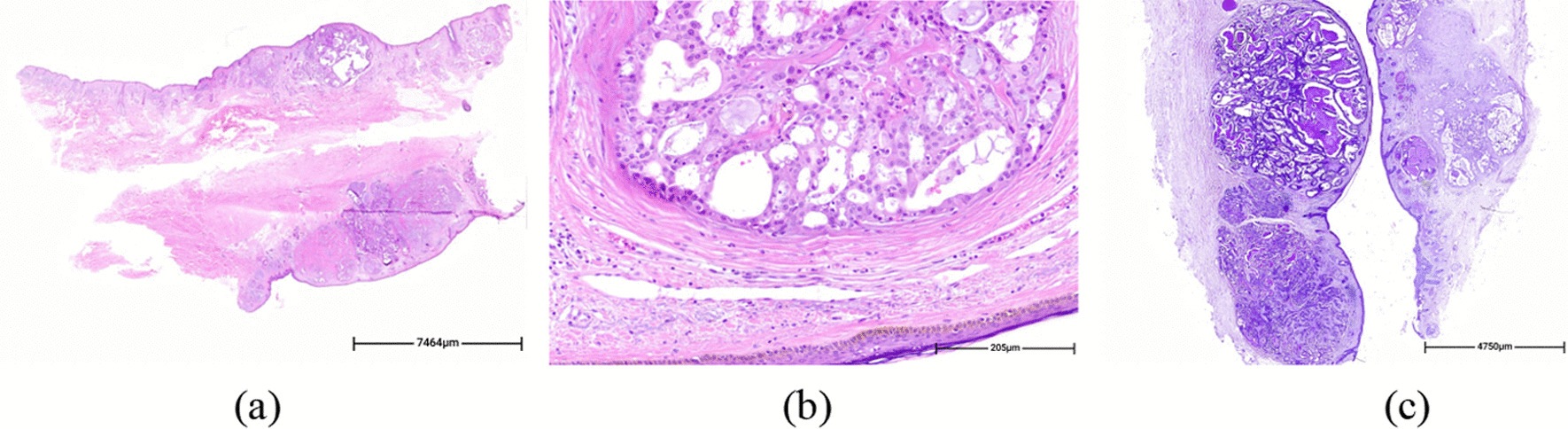
Microscopic sections revealed a dermal-based multi nodular proliferation (**a** H&E. whole-scan-view) with unremarkable interspersed dermis, underlying an uninvolved epidermis. Tumor cells have eosinophilic cytoplasm, round vesicular nuclei and small distinctive nucleoli, without high grade components (**b** H&E. ×200). PAS staining reveals PAS positive diastase resistant secretions (**c** PAS. ×100)

Histopathology sections revealed a multi-nodular proliferation of neoplastic cells infiltrating adipose-muscular tissue (Fig. 3a), with variable architectural patterns including papillary, cribriform, closely packed micro-cysts and tubules (Fig. 3b) lined by bland-looking cells with abundant colloid-like Periodic acid Schiff (PAS) positive and diastase resistant bubbly secretions (Fig. 3c). The neoplasm was associated by hemorrhagic foci, hemosiderin depositions, and cholesterol clefts with no evidence of lymph node residue. Tumor cells had eosinophilic and vacuolated cytoplasm with a high nuclear to cytoplasm (N/C) ratio, relatively monomorphic round vesicular nuclei, and small distinct nucleoli (Fig. 3d, e). Foci of HGT, including of desmoplastic stromal reaction, comedo-type necrosis, nuclear pleomorphism, and loss of secretory activity with perineural invasion were noted (Fig. 3f). Nevertheless, evaluation of the pre-auricular mass revealed a low-grade conventional SC without HGT or even neural invasion (Fig. 4a–c).

**Fig. 5:**
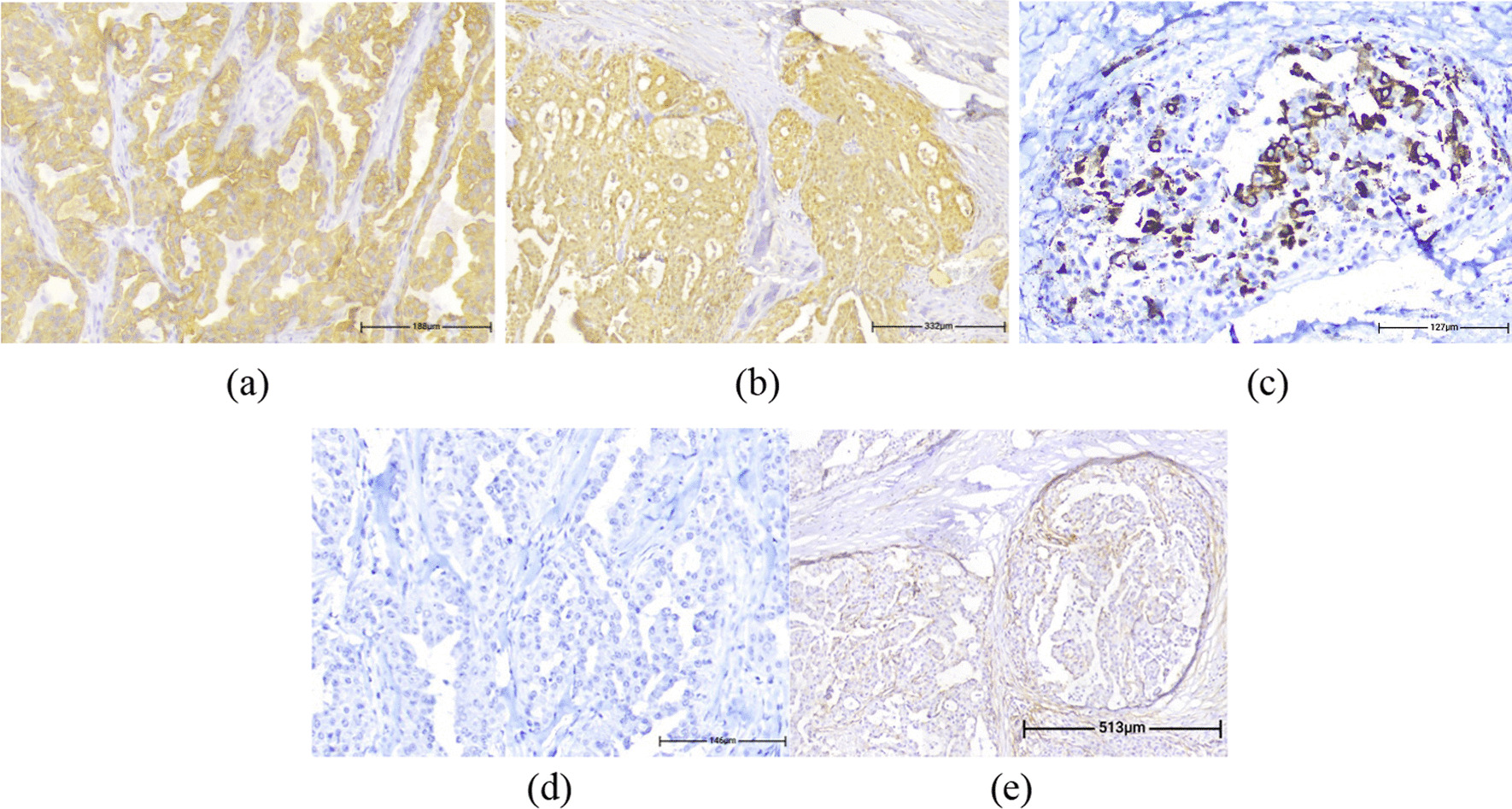
By immune staining tumoral cells are strongly positive for cytokeratin 7 (**a** IHC. ×200) and S100 (**b** IHC. ×100), patchy positive for Mammaglobin (**c** IHC. ×200) and negative for gross cystic disease fluid protein 15 (**d** IHC. ×200) and DOG1 (**e** IHC. ×100)

By immune-staining tumoral cells were strongly positive for Ck7 (Fig. 5a) and S100 (Fig. 5b), patchy positive for Mammaglobin (Fig. 5c) and GATA3, while negative for GCDFP-15 (Fig. 5d), DOG1 (Fig. 5e), ER, AR, Synaptophysin and P63. Histomorphology and immune-staining results were consistent with the final diagnosis of SC.

Unfortunately, the patient didn’t consent for adjuvant radiation therapy.12 months later throughout the follow-up, he developed a new recurrence at the cervical adipo-muscular site (level IV-V-VII) without lymph node involvement. Surgery was done again to remove the mass. However, the surgery was complicated due to thoracic duct injury, and developed a chylous fistula which needed 2-weeks of hospitalization and reoperation.

## Discussion and conclusion

SC is a recently described entity and owes its name to SC of the breast based on their histological, immunohistochemical (IHC), and genetic similarities [16, 17]. The updated WHO classification of head and neck Tumors (4th edition, 2017) substituted SC for the previous MASC designation [18]. About 642 cases until 2020 (Lisia Daltro 2020) have been reported in the literature, and the incidence of this neoplasm has been estimated for less than 0.3% of all salivary gland tumors [2].

This entity can be confused with various other low-grade salivary gland tumors such as adenocarcinoma (NOS), mucin-producing signet ring adenocarcinoma, MEC, and ACC [2–4]. The latter is the most common tumor reported incorrectly instead of SC [6, 16] because of its variable histological features including low-grade papillary-cystic, and glandular patterns, intra-luminal secretory materials, ovoid nuclei, and relatively abundant granular eosinophilic cytoplasm, corresponding to papillary-cystic or follicular types of ACC [4]. Variable architectural patterns including, papillary, cribriform, closely packed micro-cysts and tubules of our case initially suggested the diagnosis of ACC, however, the presence of PAS-positive and diastase-resistant bubbly secretions and the result of the IHC study which was strongly positive for Ck7 and S100, weakly positive for GATA3 and negative for DOG1, ER, AR, Synaptophysin, and P63, all were in favor of SC. Although ETV6-NTRK3 Gene Fusion has been used for confirmation of the SC diagnosis, many recent studies claim that morphology in conjunction with proper IHC panel is sufficient enough for definite diagnosis [8, 9].

According to the previous studies and case reports, the recommended IHC panel is a combination of S100, Mammaglobin, P63, and DOG1. Mammaglobin is much more sensitive than GCDFP-15 [4, 19, 20], so was in our case, which showed a positive reaction for Mammaglobin with no reactivity for GCDFP-15.

In addition, this case showed solid and cribriform growth patterns, atypical cytologic changes, comedo-type necrosis, and neural invasion. A review of the past articles indicates 22 and 21 SC cases with high-grade features and neural invasion respectively. Although in general, the presence of HGT predicts a poor prognosis, there are some cases without HGT that show an aggressive nature and neural invasion [21]. An argument commonly put forward is that, HGT tends to occur more in men with an older median age and greater tumor dimension [1]. There are also some studies representing IHC panels including EGFR, β-Catenin, and Cyclin-D1 for better recognition of high-grade components [3, 22].

SC mortality rate has been reported in 10 cases so far, some succeeding multiple recurrences and others after disseminated metastasis [3, 17]. To illustrate, Majeweska (2014) presented 7 SC cases, one of them showed HGT and died after 20 months following four local recurrences and distant metastasis to the lung and bone. The other one was located in the hard palate, without HGT at first presentation, and became high grade during multiple regional recurrences (died after 79 months) [23].

Cervical lymph node metastasis is relatively common in SC, counting about 31 cases up to now. 14 metastatic SC cases to the lung and pleura have also been reported (Table 1). David in 2018 presented 13 SC cases with a mean age of 54 years, one of them presented with lung metastasis four years after the initial therapy, while the patient had done well following metastasectomy (during a 5.5-year follow-up) [24]. Same as Todd et al. in 2015 who presented 14 SC cases, one with high-grade features and lung metastasis (developed after 4 years) and free of disease 3 years after metastatectomy [25]. There are also 3 reported cases of axial bone metastases (thoracic-cervical spine [14], and scapula-pelvic [15]), and only one cervical adipomuscular tissue metastasis [3].

Moreover, there are about 39 reported SC cases undergone recurrence after therapy. For instance, Baghai in 2017 described 10 SC cases, two of them presented with late recurrence (more than 10 years after the initial surgery) and two others died, one after pelvic and scapular bone metastasis and the other one died following multiple recurrences (15 years after the initial diagnosis) [15].

In conclusion, while SC is mainly indolent, some reports claim that multiple recurrences, unpredictable metastasis, and even death have been occurred in some patients. Identification and reporting the SC cases with catastrophic outcomes will indeed highlight the need for a more aggressive and watchful initial workup of these patients.

## Data Availability

Data sharing is not applicable to this article as no datasets were generated or analysed during the current study.

## Abbreviations

SC: Secretory carcinoma
WHO: World Health Organization
MASC: Mammary analogue secretory carcinoma
MEC: Mucoepidermoid carcinoma
ACC: Acinic cell carcinoma
HGT: High-Grade transformation
CT: Computed tomography
PAS: Periodic acid Schiff
N/C: Nuclear to Cytoplasm
IHC: Immunohistochemistry
